# Radioprotective effects of *Sipunculus nudus* L. polysaccharide combined with WR-2721, rhIL-11 and rhG-CSF on radiation-injured mice

**DOI:** 10.1093/jrr/rrv009

**Published:** 2015-04-06

**Authors:** Shuqi Jiang, Xianrong Shen, Yuming Liu, Ying He, Dingwen Jiang, Wei Chen

**Affiliations:** 1 The PLA Key Laboratory of Biological Effect and Medical Protection on Naval Vessel Special Environment, Department of Protection Medicine, Naval Medical Research Institute, 880 Xiangyin Road, Yangpu District, Shanghai 200433, China; 2 Zhejiang Chinese Medical University, Hangzhou 310053, China

**Keywords:** *Sipunculus nudus* L*.* polysaccharide, radioprotection, WR-2721, rhG-CSF, rhIL-11, combination administration

## Abstract

This study investigated the radioprotective effect of *Sipunculus nudus* L. polysaccharide (SNP) in combination with WR-2721, rhIL-11 and rhG-CSF on irradiated mice. A total of 70 Imprinting Control Region (ICR) mice were divided into seven groups: the control group, the model group and five administration groups. All groups, except the control group, were exposed to a 5 Gy ^60^Co γ-ray beam. Blood parameters [including white blood cell (WBC), red blood cell (RBC) and platelet counts and hemoglobin level] were assessed three days before irradiation, and the on the 3rd, 7th and 14th days after irradiation. Spleen, thymus and testicular indices, DNA contents of bone marrow cells, bone marrow nucleated cells, sperm counts, superoxide dismutase (SOD), malondialdehyde (MDA), testosterone and estradiol levels in the serum were assessed on the 14th day after irradiation. The combined administration of SNP, WR-2721, rhIL-11 and rhG-CSF exerted synergistic recovery effects on peripheral blood WBC, RBC and platelet counts and hemoglobin levels in irradiated mice, and synergistic promotion effects on spleen, thymus, testicle, bone marrow nucleated cells and sperm counts in irradiated mice. The synergistic administration increased the serum SOD activities and serum testosterone content of irradiated mice, but synergy decreased the content of serum MDA and estradiol in irradiated mice. These results suggest that the combined administration of SNP, WR-2721, rhIL-11 and rhG-CSF should increase the efficacy of these drugs for acute radiation sickness, protect immunity, hematopoiesis and the reproductive organs of irradiated-damaged mice, and improve oxidation resistance in the body.

## INTRODUCTION

Radiation affects people's lives. People who work in nuclear plants, nuclear reactors, nuclear medicine, nuclear science and nuclear weapons may receive low-dose irradiation. Nuclear accidents may expose people to high-dose irradiation. Radiotherapy is an important modality for cancer therapy, but there are major drawbacks, primarily because of the severe side effects that are generated as a result of tissue damage. Some attention has been paid to the treatment of acute radiation sickness. Several types of antiradiation drugs have been developed, such as WR-2721 (amifostine), estrogen, cytokines, natural antioxidants and immune stimulators. However, the inherent side effects of these antiradiation drugs and their limited pharmacodynamic action create the need for further research for new drugs and new therapy strategies, including combination administration, which can be advantageous.

Previous studies have demonstrated that WR-2721 administration for head and neck cancer is associated with a high rate of serious adverse effects, including hypotension, vomiting and allergic reactions [1]. Some findings have suggested that the risk of life-threatening cutaneous adverse reactions to WR-2721 by the dose 200 mg/m^2^ and 250 mg/m^2^ for 15–42 days could increase significantly during radiotherapy and/or chemotherapy [2]. Current anti-radiation drug research has increasingly focused on cytokines. Clinical studies indicate that the treatment of severe chronic neutropenia with rhG-CSF stimulates bone marrow production and neutrophil maturation, which thus increases circulating neutrophils and reduces infection-related events [3]. rhIL-11 has potent *in vivo* thrombopoietic effects in normal and myelosuppressed non-human primates, and rhIL-11 may be an important therapy for reducing the severity and duration of thrombocytopenia resulting from chemotherapy [4]. Cytokines in combination have been used as a primary therapeutic measure for hematopoietic system radiation damage in experimental and clinical research [5, 6]. The combination of rhG-CSF and rhIL-11 for antiradiation treatment has been reported to achieve satisfactory effects [7, 8]. *Sipunculus nudus* L*.* polysaccharide (SNP) is a bioactive polysaccharide that is extracted from *Sipunculus nudus* L. We reported previously that oral SNP administration in beagle dogs facilitated the recovery of hematopoietic bone marrow damage induced by γ-radiation [9]. Extracts of *Sipunculus nudus* L*.* exhibit some physiological functions, such as antiradiation, anti-oxidation, antisenescence and antifatigue, and improve immunity [10–15]. Recently, SNP was demonstrated to have antioxidant activity and to improve immunity, which may play an important role in the treatment of irradiation damage.

## MATERIALS AND METHODS

### Materials

*Sipunculus nudus* L. was purchased from Beihai (Guangxi, China); it was identified by Professor Jia Fuxing from the Naval Medical Research Institute of the PLA. The SNP was prepared in our laboratory as described in a previous study [16], and the polysaccharide content of the SNP was 87%. WR-2721 was purchased from Hubei Gedian Humanwell Pharmaceutical Co. Ltd (Ezhou, Hubei, China). rhIL-11 was purchased from Beijing SL Pharmaceutical Co. Ltd (Beijing, China). rhG-CSF was purchased from Shanghai Sunway Biotech Co. Ltd (Shanghai, China). Superoxide dismutase (SOD), and malondialdehyde (MDA) kits were purchased from the Nanjing Jiancheng Bioengineering Institute (Nanjing, Jiangsu, China). Testosterone and estradiol kits were purchased from R&D Systems, China Co. Ltd (Shanghai, China).

### Animals and treatments

Three-week-old male imprinting control region (ICR) mice, weighing 17–19 g, were purchased from the Sino–British SIPPR/BK Laboratory. Animal Co. Ltd mice were maintained under conditions of standard lighting (12:12 h light:dark cycle), temperature (23–27°C) and humidity (50–60%) with freely available food and water. The Institutional Animal Care and Use Committees of the Naval Medical Research Institute approved all procedures for animal care and treatment.

The animals were randomly divided into seven groups of 10 mice each. The administration techniques and doses for each group are shown in Table 1.

**Table 1.** Administration techniques and doses for each group of ICR mice

Group	Administration and dose
Control group	0.2 ml/0.02 kg H_2_O intragastrically once daily
Model group	0.2 ml/0.02 kg H_2_O intragastrically once daily
W group	400 mg/kg WR-2721 intraperitoneally 30 min before irradiation
S + I + G group	400 mg/kg SNP intragastrically once daily, and 200 μg/kg rhIL-11 and 30 μg/kg rhG-CSF subcutaneously once daily for 7 days after irradiation
S + W group	400 mg/kg SNP intragastrically once daily, and 400 mg/kg WR-2721 intraperitoneally 30 min before irradiation
W + I + G group	400 mg/kg WR-2721 intraperitoneally 30 min before irradiation, and 200 μg/kg rhIL-11 and 30 μg/kg rhG-CSF subcutaneously once daily for 7 days after irradiation
S + W + I + G group	400 mg/kg SNP intragastrically once daily, and 400 mg/kg WR-2721 intraperitoneally 30 min before irradiation, and 200 μg/kg rhIL-11 and 30 μg/kg rhG-CSF subcutaneously once daily for 7 days after irradiation

### Animal irradiation

Animals in all groups, except the control group, were whole-body exposed to 5.0 Gy of ^60^Co γ- radiation (at the Radiation Center of the Second Military Medical University, Shanghai, China) in a specially designed, well-ventilated acrylic box at a dose rate of 0.8 Gy/min 30 min after WR-2721 administration on the 8th day. Animals were sacrificed via cervical dislocation on the 14th day after irradiation, according to the experimental schedule.

### Peripheral blood cell count

Whole blood was collected from the mouse tail into EDTA-coated tubes three days before irradiation and on the 3rd, 7th and 14th days after irradiation. Peripheral blood cell counts were obtained using a veterinary hematology analyser (Nihon Kohden Corporation, Japan) according to the operator's manual.

### Organ weight index detection

Animals were sacrificed on the 14th day after irradiation. The spleen, thymus and testis were separated, and organ indices were determined using the following equation:

Organ index=organ weight/bodyweight×1000.

### Bone marrow DNA content detection

Animals in all groups were sacrificed 14 days after irradiation. The right femur of each animal was removed and cleaned of adherent tissues. The bone marrow was flushed into 5 ml of a 5-mmol/l CaCl_2_ solution and incubated at 4°C for 30 min. Cell suspensions were centrifuged at 2500 rpm for 15 min, and the pellet was resuspended in 5 ml of a 0.2-mol/l HClO_4_ solution. The suspension was incubated at 90°C for 15 min and filtered through a 0.22-μm membrane. The absorbance at 260 nm was detected using a 759-UV spectrophotometer (Shanghai APL instrument Co. Ltd).

### Detection of bone marrow nucleated cells

The left femur of each animal was removed 14 days after irradiation, and the bone marrow was flushed into 2 ml of physiological saline containing 20-U/ml heparin. A total of 0.39 ml of 2% acetic acid was added to 10 μl of the cell suspension, and the number of nucleated cells was counted using a cell counter.

### Sperm count

The left parorchis of each animal was removed 14 days after irradiation, and sperm was flushed into 3 ml of physiological saline. Sperm was quantified under a microscope after a 15-min incubation at 37°C.

### Detection of SOD, MDA, testosterone and estradiol in serum

Blood was collected from an eye socket vein into clean centrifuge tubes. Serum was prepared by centrifugation at 3000*g* for 15 min. SOD and MDA were determined using kits from Nanjing Jiancheng Bioengineering Institute according to the manufacturer's instructions, and testosterone and estradiol were determined using ELISA kits from R&D Systems China Co. Ltd.

### Statistical analysis

Data are presented as means ± standard deviation (SD). The unpaired Student's *t* test was performed for statistical analyses. The analysis was performed using the SAS software package.

## RESULTS

### Effect of the combination of SNP, WR-2721, rhIL-11 and rhG-CSF on peripheral blood WBCs of irradiated mice

The results are presented in Table 2. The WBCs did not significantly differ between groups three days before irradiation (*P* > 0.05). However, WBCs in all irradiated groups decreased significantly from the 3rd to the 14th day after irradiation compared with the control group (*P* < 0.01). WBCs in the W, S + W, W + I + G and S + W + I + G groups increased significantly on the 3rd and 7th days after irradiation compared with the model group (*P* < 0.05 or *P* < 0.01). WBCs in the S + W + I + G group increased significantly on the 14th day after irradiation compared with the model group (*P* < 0.01). The S + W + I + G group had the highest elevation of WBCs in the irradiated mice. These results suggest that the combination of SNP, WR-2721, rhIL-11 and rhG-CSF produced a synergistic promotion effect on peripheral blood WBCs in irradiated mice.

**Table 2.** Peripheral blood WBC counts in experimental mice (mean ± SD, × 10^9^·l^−1^)

Group	3 Days before IR	Day 3 after IR	Day 7 after IR	Day 14 after IR
Control group	7.1 ± 1.2	11.3 ± 1.6	17.0 ± 4.5	14.8 ± 3.9
Model group	7.2 ± 0.9	1.3 ± 0.3^●^	2.5 ± 1.1^●^	5.5 ± 1.6^●^
W group	7.4 ± 1.2	2.1 ± 0.8^●^*	5.0 ± 2.2^●Δ^	6.5 ± 1.7^●^
S + I + G group	7.0 ± 1.0	1.6 ± 0.3^●^	1.9 ± 1.1^●^	6.9 ± 2.9^●^
S + W group	7.2 ± 1.0	2.3 ± 0.7^●Δ^	5.1 ± 2.9^●^*	4.6 ± 1.0^●^
W + I + G group	7.4 ± 1.3	2.7 ± 0.9^●Δ^	5.3 ± 2.0^●Δ^	5.3 ± 1.5^●^
S + W + I + G group	7.2 ± 0.9	3.0 ± 0.5^●Δ^	7.9 ± 2.6^●Δ^	9.9 ± 1.4^●Δ^

Compared with the control group: closed circle: *P* < 0.01. Compared with the model group: asterisk: *P* < 0.05, closed triangle: *P* < 0.01.

### Effect of the combination of SNP, WR-2721, rhIL-11 and rhG-CSF on peripheral blood platelets

Table 3 shows that platelets in none of the irradiated groups were significantly decreased three days before irradiation or on the third day after irradiation compared with the control group (*P* > 0.05). Platelets in all irradiated groups decreased significantly on the 7th day after irradiation compared with the control group (*P* < 0.01). Platelets in the W + I + G and S + W + I + G groups increased significantly on the 7th day after irradiation compared with the model group (*P* < 0.05 or *P* < 0.01). Platelets in the model, W, S + W and W + I + G groups decreased significantly on the 14th day after irradiation compared with the control group (*P* < 0.05 or *P* < 0.01). Platelets in the S + W + I + G group returned to normal levels on the 14th day after irradiation. These results suggest that irradiation decreases the level of peripheral blood platelets 7 days after irradiation, and that the combined administration of SNP, WR-2721, rhIL-11 and rhG-CSF effectively restores platelet levels.

**Table 3.** Peripheral blood platelet counts in experimental mice (mean ± SD, × 10^9^·l^−1^)

Group	3 days before IR	Day 3 after IR	Day 7 after IR	Day 14 after IR
Control group	994 ± 104	871 ± 100	954 ± 128	1119 ± 137
Model group	1032 ± 91	933 ± 116	459 ± 93^●^	810 ± 156^●^
W group	987 ± 87	998 ± 105^○^	545 ± 136^●^	841 ± 131^●^
S + I + G group	1029 ± 102	1167 ± 171^●Δ^	381 ± 84^●^	1016 ± 265
S + W group	1076 ± 127	904 ± 143	487 ± 95^●^	981 ± 112^○^
W + I + G group	1044 ± 145	872 ± 87	544 ± 75^●^*	944 ± 124^○^
S + W + I + G group	1128 ± 97	968 ± 155	579 ± 74^●Δ^	1000 ± 201*

Compared with the control group: open circle: *P* < 0.05, closed circle: *P* < 0.01. Compared with the model group: asterisk: *P* < 0.05, closed triangle: *P* < 0.01.

### Effect of the combination of SNP, WR-2721, rhIL-11 and rhG-CSF on peripheral blood RBCs

Table 4 shows that peripheral blood RBC levels in all groups were not significantly decreased three days before irradiation or on the third day after irradiation (*P* > 0.05). RBC counts in the model, W, S + I + G and W + I + G groups decreased significantly on the 7th day after irradiation compared with the control group (*P* < 0.05 or *P* < 0.01). RBCs in the S + W + I + G group returned to normal levels. RBCs in the model, W, S + I + G and S + W groups decreased significantly on the 14th day after irradiation compared with the control group (*P* < 0.05 or *P* < 0.01). RBCs in the S + W + I + G and W + I + G groups returned to normal levels. These results suggest that irradiation decreases the level of peripheral blood RBCs 7 days after irradiation, and that the combined administrations of SNP, WR-2721, rhIL-11, rhG-CSF and the combination of WR-2721, rhIL-11, rhG-CSF exhibit a synergistic promotion effect on peripheral blood RBCs in irradiated mice.

**Table 4.** Peripheral blood RBC counts in experimental mice (mean ± SD, × 10^12^·l^−1^)

Group	3 Days before IR	Day 3 after IR	Day 7 after IR	Day 14 after IR
Control group	8.28 ± 0.39	7.70 ± 0.94	7.83 ± 0.71	8.70 ± 0.95
Model group	8.30 ± 0.59	8.56 ± 0.80	6.93 ± 0.93^○^	6.17 ± 1.31^●^
W group	8.39 ± 0.33	8.50 ± 0.92	7.03 ± 0.79^○^	7.56 ± 0.79^○^*
S + I + G group	8.69 ± 0.52	8.29 ± 0.84	5.94 ± 0.88^●^*	5.73 ± 0.89^●^
S + W group	8.95 ± 0.77	8.64 ± 1.03	7.00 ± 1.77	7.30 ± 0.59^●^*
W + I + G group	8.57 ± 0.37	8.28 ± 0.77	6.92 ± 0.95^○^	8.42 ± 1.26^Δ^
S + W + I + G group	9.01 ± 0.28	8.32 ± 0.71	7.15 ± 0.98	8.24 ± 0.74^Δ^

Compared with the control group: open circle: *P* < 0.05, closed circle: *P* < 0.01. Compared with the model group: asterisk: *P* < 0.05, closed triangle: *P* < 0.01.

### Effect of the combination of SNP, WR-2721, rhIL-11 and rhG-CSF on peripheral blood hemoglobin

Table 5 shows that peripheral blood hemoglobin in the irradiated groups decreased significantly on the 7th day after irradiation compared with the control group (*P* < 0.05 or *P* < 0.01). The hemoglobin levels in the W, S + W, W + I + G and S + W + I + G groups increased significantly on the 14th day after irradiation compared with the model group (*P* < 0.01). These results suggest that the combined administrations of SNP, WR-2721, rhIL-11 and rhG-CSF exhibit a synergistic promotion effect on peripheral blood hemoglobin in irradiated mice.

**Table 5.** Peripheral blood hemoglobin in experimental mice (mean ± SD, g·L^−1^)

Group	3 Days before IR	Day 3 after IR	Day 7 after IR	Day 14 after IR
Control group	114 ± 9	111 ± 14	111 ± 7	126 ± 9
Model group	117 ± 9	118 ± 9	93 ± 14^●^	87 ± 18^●^
W group	115 ± 8	119 ± 12	98 ± 14^○^	109 ± 8^●Δ^
S + I + G group	114 ± 9	107 ± 9*	79 ± 11^●^*	84 ± 11^●^
S + W group	114 ± 12	125 ± 24	94 ± 24^○^	111 ± 10^●Δ^
W + I + G group	112 ± 8	121 ± 6	99 ± 13^○^	123 ± 6^Δ^
S + W + I + G group	114 ± 9	123 ± 8^○^	89 ± 19^●^	116 ± 3^●Δ^

Compared with the control group: open circle: *P* < 0.05, closed circle: *P* < 0.01. Compared with the model group: asterisk: *P* < 0.05, closed triangle: *P* < 0.01.

### Effect of the combination of SNP, WR-2721, rhIL-11 and rhG-CSF on organ indices

Table 6 shows that the spleen indices of the treated groups did not decrease significantly on the 14th day after irradiation compared with the control group (*P* > 0.05). The thymus indices of the model, W, S + I + G and S + W groups decreased significantly compared with the control group (*P* < 0.05 or *P* < 0.01). The thymus indices of the S + W, W + I + G and S + W + I + G groups increased significantly compared with the model group (*P* < 0.05 or *P* < 0.01). The testicle indices of the model, W and S + I + G groups decreased significantly compared with the control group (*P* < 0.01), and the testicle indices of the S + I + G, S + W, W + I + G and S + W + I + G groups increased significantly compared with the model group (*P* < 0.05 or *P* < 0.01). These results suggest that irradiation has no effect on spleen, but that it damages the thymus and testicles, and that the combined administration of SNP, WR-2721, rhIL-11 and rhG-CSF alleviates thymus and testicular injury in irradiated mice.

**Table 6.** Organ indices in experimental mice (mean ± SD)

Group	Spleen index	Thymus index	Testicle index
Control group	5.53 ± 2.42	1.90 ± 0.40	3.10 ± 0.69
Model group	6.48 ± 2.42	1.21 ± 0.27^●^	1.99 ± 0.16^●^
W group	6.56 ± 1.94	1.43 ± 0.23^○^	2.08 ± 0.31^●^
S + I + G group	11.45 ± 2.54^●Δ^	1.28 ± 0.32^●^	2.36 ± 0.26^●^*
S + W group	5.87 ± 1.99	1.48 ± 0.24^○^*	2.64 ± 0.38^Δ^
W + I + G group	6.71 ± 1.93	1.66 ± 0.24^Δ^	2.54 ± 0.24^Δ^
S + W + I + G group	4.89 ± 1.49	1.87 ± 0.35^Δ^	2.64 ± 0.29^Δ^

Compared with the control group: open circle: *P* < 0.05, closed circle: *P* < 0.01. Compared with the model group: asterisk: *P* < 0.05, closed triangle: *P* < 0.01.

### Effect of the combination of SNP, WR-2721, rhIL-11 and rhG-CSF on the DNA content of bone marrow cells

**Fig. 1.**
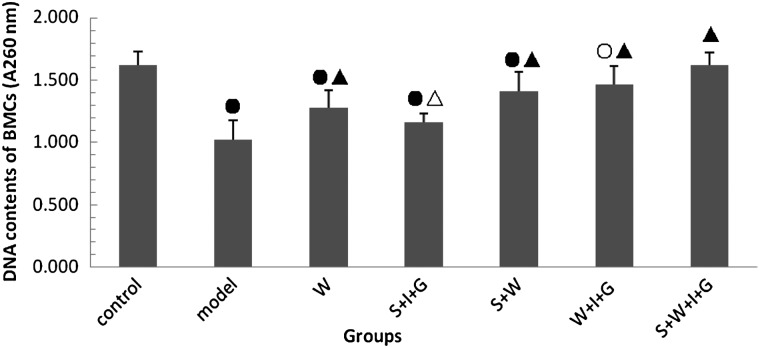
DNA contents of bone marrow cells in experimental mice. Compared with the control group: open circle: *P* < 0.05, closed circle: *P* < 0.01. Compared with the model group: open triangle: *P* < 0.05, closed triangle: *P* < 0.01.

Figure 1 shows that the DNA contents of bone marrow cells decreased significantly in all irradiated groups, except the S + W + I + G group, on the 14th day after irradiation compared with the control group (*P* < 0.01). The DNA contents of the five administered groups increased significantly compared with the model group (*P* < 0.05 or *P* < 0.01), and the DNA contents of the S + W + I + G group returned to control levels (*P* > 0.05). These results suggest bone marrow cells were damaged during irradiation, and that all administration combinations ameliorated the damage. The combined administration of SNP, WR-2721, rhIL-11 and rhG-CSF produced the most improvement in the DNA contents of the bone marrow cells in the irradiated mice.

### Effect of the combination of SNP, WR-2721, rhIL-11 and rhG-CSF on bone marrow nucleated cells

**Fig. 2.**
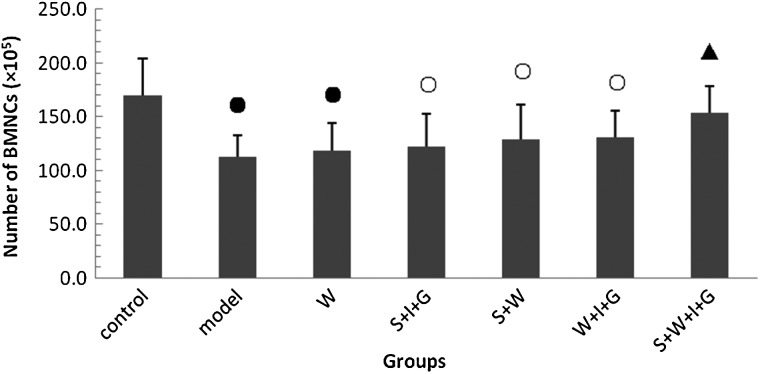
The number of bone marrow nucleated cells in experimental mice. Compared with the control group: open circle: *P* < 0.05; closed circle: *P* < 0.01. Compared with the model group: closed triangle: *P* < 0.01.

Figure 2 shows that the number of bone marrow nucleated cells (BMNCs) in all irradiated groups, except the S + W + I + G group, decreased significantly on the 14th day after irradiation compared with the control group (*P* < 0.05 or *P* < 0.01). BMNCs in the S + W + I + G group increased significantly compared with the model group (*P* < 0.01). These results suggest that the combined administration of SNP, WR-2721, rhIL-11 and rhG-CSF produced a synergistic protection effect on BMNCs in irradiated mice.

### Effect of the combination of SNP, WR-2721, rhIL-11 and rhG-CSF on sperm counts

**Fig. 3.**
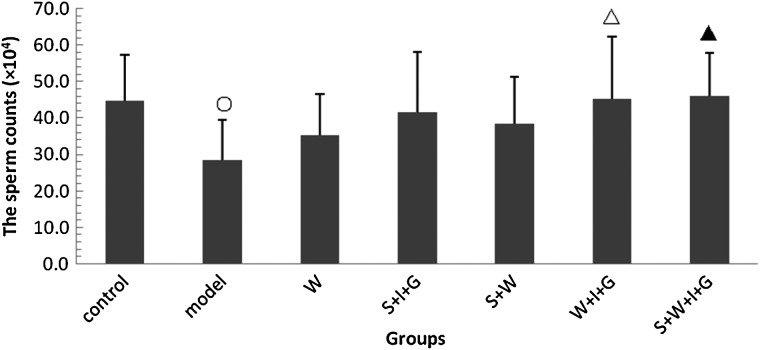
Sperm counts in experimental mice. Compared with the control group: open circle: *P* < 0.05. Compared with the model group: open triangle: *P* < 0.05, closed triangle: *P* < 0.01.

Figure 3 shows that sperm counts in the model group decreased significantly on the 14th day after irradiation compared with the control group (*P* < 0.05). Sperm counts in the W + I + G and S + W + I + G groups increased significantly compared with the model group (*P* < 0.05 or *P* < 0.01) and returned to normal levels. These results suggest that the combination of SNP, WR-2721, rhIL-11 and rhG-CSF produced a synergistic restoration effect on sperm counts in irradiated mice.

### Effect of the combination of SNP, WR-2721, rhIL-11 and rhG-CSF on serum testosterone and estradiol

The results are shown in Table 7. Serum testosterone content in the model group decreased significantly on the 14th day after irradiation compared with the control group (*P* < 0.01). The serum testosterone content of all treated groups, except the W + I + G group, increased significantly compared with the model group (*P* < 0.05 or *P* < 0.01). The testosterone level in the S + W + I + G group was significantly higher than in the control group (*P* < 0.01). These results suggest that the combinations of SNP, WR-2721, rhIL-11 and rh-CSF synergistically improved serum testosterone content in irradiated mice.

**Table 7.** Serum testosterone and estradiol content in experimental mice (mean ± SD)

Group	Testosterone content (ng·ml^−1^)	Estradiol content (pmol·l^−1^)
Control group	4.8 ± 0.4	12.0 ± 2.3
Model group	3.7 ± 0.6^●^	26.8 ± 2.5^●^
W group	4.5 ± 0.8*	15.6 ± 3.9^○Δ^
S + I + G group	4.6 ± 0.7*	13.7 ± 2.8^Δ^
S + W group	5.4 ± 1.1^Δ^	13.2 ± 3.9^Δ^
W + I + G group	4.3 ± 1.2	12.9 ± 2.5^Δ^
S + W + I + G group	6.9 ± 1.5^●Δ^	9.2 ± 1.1^●Δ^

Compared with the control group: open circle: *P* < 0.05, closed circle: *P* < 0.01. Compared with the model group: asterisk: *P* < 0.05, closed triangle: *P* < 0.01.

Serum estradiol content in the model group was significantly higher than in the control group (*P* < 0.01). Serum estradiol contents of all treated groups decreased significantly compared with that of the model group (*P* < 0.01), and the estradiol level in the S + W + I + G group was lower than in the control group (*P* < 0.01). These results suggest that the various combinations of SNP, WR-2721, rhIL-11 and rh-CSF exerted a synergistic regulation effect on serum estradiol contents in irradiated mice.

### Effect of the combination of SNP, WR-2721, rhIL-11 and rhG-CSF on serum SOD activity and MDA content

The results are shown in Table 8. Serum SOD activities of all irradiated groups, except the S + W + I + G group, decreased significantly on the 14th day after irradiation compared with the control group (*P* < 0.01). SOD activities of all treated groups, except for the S + I + G group, increased significantly compared with that of the model group (*P* < 0.01). SOD activity in the S + W + I + G group was higher than in the control group (*P* < 0.01). These results suggest that the combination of SNP, WR-2721, rhIL-11 and rh-CSF synergistically improved serum SOD activity in irradiated mice.

**Table 8.** Serum SOD and MDA in experimental mice (mean ± SD)

Group	SOD (U·ml^−1^)	MDA (nmol·ml^−1^)
Control group	117.2 ± 9.2	7.01 ± 0.89
Model group	79.5 ± 7.8^●^	8.78 ± 0.92^●^
W group	92.2 ± 6.9^●Δ^	8.11 ± 0.44^●^
S + I + G group	87.8 ± 8.7^●^	8.56 ± 1.08^○^
S + W group	99.0 ± 6.6^●Δ^	8.08 ± 0.93^○^
W + I + G group	98.7 ± 11.1^●Δ^	7.89 ± 0.67^○^*
S + W + I + G group	121.4 ± 8.4^Δ^	7.49 ± 0.90^Δ^

Compared with the control group: open circle: *P* < 0.05, closed circle: *P* < 0.01. Compared with the model group: open triangle: *P* < 0.05, closed triangle: *P* < 0.01.

Serum MDA contents in all irradiated groups, except for the S + W + I + G group, increased significantly compared with the control group (*P* < 0.05 or *P* < 0.01). Serum MDA contents of the W + I + G and S + W + I + G groups decreased significantly compared with the model group (*P* < 0.05 or *P* < 0.01), and MDA content in the S + W + I + G group returned to normal levels (*P* > 0.05 compared with the control group). These results suggest that the combination of SNP, WR-2721, rhIL-11 and rhG-CSF synergistically reduced serum MDA in irradiated mice.

## DISCUSSION

The main injuries of acute radiation sickness involve the hematopoietic, reproductive and gastrointestinal systems. The primary pathological changes in bone marrow–type acute radiation sickness are suppression of bone marrow hematopoietic functions, decrease in peripheral blood WBCs and platelets, infection and hemorrhage caused by acute radiation syndrome [7]. Immunomodulation and antioxidation are two important treatments for acute radiation sickness.

*Sipunculus nudus* L. has been used as a traditional Chinese medicine in folk remedies for the treatment of tuberculosis and nocturia because this plant regulates stomach and spleen functions. Decoctions of *Sipunculus nudus* L. are traditionally used to restore health in debilities caused by various pathogens and aging. Zhang C-X reported that the polysaccharide extracted from *Sipunculus nudus* L. has potent immunostimulating and anti-hypoxia activity [10, 15]. WR-2721 was first identified by the US army's anti-radiation drug-screening program in the 1950 s, and it was approved by the FDA as a potent active radioprotectant. However, WR-2721 has inherent toxicity when administered at high cytoprotective doses. The side effects of WR-2721 include upper and lower gastrointestinal disturbances, adverse behavioral responses, and decreased performance [16]. G-CSF is a hematopoietic growth factor that stimulates the proliferation of myeloid lineage progenitor cells and granulocyte recovery and increases the survival rate of animals that receive a lethal dose of radiation. Sureda reported that recombinant human G-CSF significantly improved the survival of lethally irradiated mice [17]. IL-11 is a potent anti-inflammatory cytokine that exhibits hematopoietic growth factor activity and cytoprotective effects. rhIL-11 is used to treat thrombocytopenia and inflammatory bowel disease. rhIL-11 likely increases crypt cell survival rate and improves intestinal mucosal injury recovery after total body irradiation. Boerma found that local rhIL-11 administration ameliorated early intestinal radiation injury and reduced manifestations of intestinal radiation injury in the clinic [18].

The results of the current study demonstrated that peripheral blood WBC, RBC and platelet counts in the S + W + I + G group were most significantly ameliorated compared with the other irradiated groups. The spleen, which is the largest secondary immune organ in the body, initiates immune reactions to blood-borne antigens, and it is the site of lymphocyte growth, division and differentiation [19]. The spleen plays an important role in hematopoiesis and immune functions, and it is sensitive to irradiation [20, 21].

The main function of the thymus is to culture various T lymphocytes. The thymus regulates immune functions via stromal cell secretion of thymus hormone and cytokines [22]. Irradiation may cause spleen and thymus cell necrosis and apoptosis and reduce the volume and weight of the spleen and thymus. Spleen and thymus indices reflect the structure and function of the spleen and thymus [23]. The thymus index in the S + W + I + G group was higher than in the other groups. Bone marrow is very sensitive to irradiation. Bone marrow nucleated cells decreased after irradiation, and their DNA content was reduced. However, bone marrow micronucleated cells increased [24]. Bone marrow nucleated cells and DNA content in the model group in the present study decreased significantly, but these values were higher in the S + W + I + G group than in the other groups.

Previous studies showed that irradiation reduced the testicular index in rats, decreased sperm counts, changed sex hormone levels in serum, and caused sexual dysfunction [25]. The present study demonstrated that the testicular index, sperm counts and serum testosterone content in the S + W + I + G group were significantly higher than in the model groups (*P* < 0.01) and higher than in the other irradiated groups. Serum estradiol levels in the S + W + I + G group were significantly lower than in the model group (*P* < 0.01) and lower than in the other irradiated groups.

Irradiation produces oxygen-free radicals, which cause lipid peroxidation and large amounts of abnormal metabolic material. Serum SOD and MDA in the model group differed significantly from those in the normal group (*P* < 0.01). The effects of the combination of SNP, WR-2721, rhIL-11 and rhG-CSF on serum SOD and MDA were better than other administrations. These results suggest that the combined administration of SNP, WR-2721, rhIL-11 and rhG-CSF exerts a significant synergistic promotion effect on the peripheral hemogram, and a significant synergistic protective effect against thymus, testicle and bone marrow damage in irradiated mice. The combined administration of SNP, WR-2721, rhIL-11 and rhG-CSF protected against immune, hematopoietic, and reproductive organ damage in irradiated mice and improved antioxidation in the body. This combination treatment may be feasible as a new strategy for the clinical treatment of acute radiation sickness.

## FUNDING

This work was supported by National Science and Technology Major Project of China (No. 2008ZXJ09004-027). Funding to pay the Open Access publication charges for this article was provided by National Science and Technology Major Project of China (No. 2008ZXJ09004-027).
